# New Aspects of Silibinin Stereoisomers and their 3-O-galloyl Derivatives on Cytotoxicity and Ceramide Metabolism in Hep G_2_ hepatocarcinoma Cell Line

**Published:** 2016

**Authors:** Mahdi Mashhadi Akbar Boojar, Mahsa Hassanipour, Shahram Ejtemaei Mehr, Masoud Mashhadi Akbar Boojar, Ahmad Reza Dehpour

**Affiliations:** a *Department of Pharmacology, School of Medicine, Tehran University of Medical Sciences, Tehran, Iran.*; b *Experimental Medicine Research Center, Tehran University of Medical Sciences, Tehran, Iran. *; c *Department of Physiology and Pharmacology, School of Medicine, Rafsanjan University of Medical Sciences, Kerman, Iran.*; d *Department of Cell and Molecular Biology, Faculty of Biological Sciences, Kharazmi University, Tehran, Iran.*

**Keywords:** Ceramidase, Silymarin, Sphingomyelinase, Glucosyl ceramide synthase, Ceramide

## Abstract

Ceramide as a second messenger is a key regulator in apoptosis and cytotoxicity. Ceramide-metabolizing enzymes are ideal target in cancer chemo-preventive studies. Neutral sphingomyelinase (NSMase), acid ceramidase (ACDase) and glucosyl ceramide synthase (GCS) are the main enzymes in ceramide metabolism. Silymarin flavonolignans are potent apoptosis inducers and silibinin is the most active component of silymarin. This study evaluated the effects of silybin A, silybin B and their 3-O-gallyl derivatives (SGA and SGB) at different concentrations (0-200 micro molar) on ceramide metabolism enzymes in Hep G2 hepatocarcinoma cell line. Cell viability, caspase-3 and 9 activities, total cell ceramide and the activities of ACDase, NSMase and GCS were evaluated. Under silibinin derivatives treatments, cell viability decreased and the activities of caspase-3 and 9 increased in a dose dependent manner among which SGB was the most effective one (P<0.05). Total cell ceramide and the activity of NSMase, the enzyme which elevates ceramide level, increased by silibinin derivatives. Furthermore, the activities of removing ceramide enzymes (ACDase and GCS) decreased efficiently. The galloyl esterification increased the activity of silibinin isomers. Consequently, this study reveals new sibilinin effects on ceramide metabolism and potential strategies to enhance the antineoplastic properties of this compound.

## Introduction

Polyphenolic flavonoids are one of the most abundant groups of phytochemical compounds and are present widely in a broad range of fruits and vegetables (1). Flavonoids possess antioxidant, anticarcinogens and antiproliferative properties and have been utilized for the treatment of several diseases, particularly in cancers (2, 3). 

Silymarin as a flavonoid is a popular dietary supplement isolated from the seeds of *Silybum marianum* (L.) Gaertn (Family Asteraceae), known as milk thistle in worldwide. Its flavolignan derivatives are used as complementary and alternative treatments for hepatocellular carcinoma and other neoplastic tumors in recent decades (4, 5). Furthermore, it has been used in the prevention and treatment of viral hepatitis, cirrhosis caused by alcohol abuse and liver damage caused by medications or industrial toxins in folk and modern medicine (6, 7).

Apart from strong antioxidant effect, free radical trapping properties and preventive effect on lipid peroxidation, it has been known as a remarkable anti-cancer agent (8). Its chemo-preventive efficacy has been demonstrated in pre-clinical cell culture and animal studies in several types of cancers including epithelial, bladder, colon, prostate, lung and ovary (9-12). Silymarin is also a potent inducer of apoptosis by increasing the expression of the proapoptotic protein such as Bax, p53 and decreasing antiapoptotic proteins Bcl-2 and Bcl-xl (13, 14).

Silymarin extract is mainly composed of four flavonolignan isomers, namely silibinin, isosilybin, silydianin and silychristin. Among these isomers, sibilinin is the most active and major component (about 60-70% of silymarin). It occurs in two diastereoisomeric flavonolignan forms: silybin A (SA) (2R, 3R, 10R, 11R) and B (2R, 3R, 10S, 11S) (SB) in 1:1 ratio (15, 16). 

Ceramide is contained in sphingolipids, which are important integral components of cell membranes. Ceramide is produced via acylation of free primary amine group of sphingoid bases and possesses important bioactive properties. Recent studies have focused on ceramide roles in cellular metabolism under stress conditions and in response to therapeutic agents (17). The biosynthesis and degradation of ceramide are regulated by several enzymes that their activities may alter ceramide contents within the cell (18).

The first stage of ceramide *de novo* biosynthesis pathway is condensation of serine and palmitoyl-CoA in a one-way reaction by serine palmitoyl transferase (SPT), which is targeted by many anti-neoplastic drugs such as daunorubicin and etoposide for cancer chemotherapy (19-21).

Neutral sphingomyelinase (NSMase) is an important enzyme in ceramide metabolism that cleaves sphingomyeline to ceramide and phosphatidyl choline by a reversible reaction. This enzyme occupies a considerable position on molecular biology through ceramide formation that leads to cell death raised from apoptosis induction (22).

Acid ceramidase (ACDase) is a lysosomal enzyme which splits ceramide into sphingosine and fatty acid. The high ACDase expression is not only involved in carcinogenesis, but it also confers resistance to radiotherapy and chemotherapy (23, 24). Accordingly, inhibitors of ACDase have been employed to enhance the cytotoxic effects of chemotherapy drugs in different tumor cell lines (25-27).

Ceramide can undergo glycosylation by glucosyl ceramide synthase (GCS) which catalyzes the first step to form glycosyl ceramide and subsequently glycosphingolipids (28). The function of this enzyme in the ceramide degradation represented as a resistant factor against the induced apoptosis by tumor necrosis factor (TNF) (29). 

Depending on the preventive and therapeutic efficacy of silibinin against cancer, suitable chemical modifications on its structure to achieve a more effective compound is valuable. It also has been shown that 3-O-galloyl substitution of flavonoids such as sibilinin may lead to new biological activities and improve the pharmacological potency (30). Furthermore, since the poor water solubility of silibinin decreases its efficiency at tumor sites, structural changes for increasing polarity and consequently hydrosolubility of the molecule could be helpful to potentiate its therapeutic effects (31).

**Figure 1:**
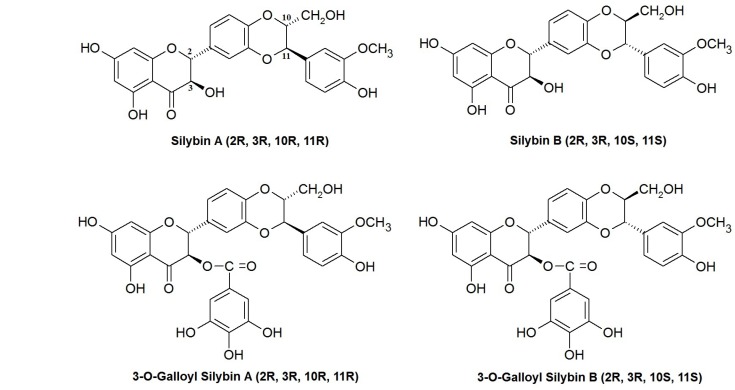
Chemical structures of silibinin stereoisomers and their 3-O-galloyl derivatives assessed in this study.

Regarding the crucial role of ceramide and related pathways on cell viability and apoptosis, studying the anti-cancer activity of silibinin stereoisomers in relation with the enzymes associated with ceramide metabolism seems to be necessary. In this study, we evaluated the effects of four silibinin derivatives silybin A (SA), silybin B (SB) and their 3-O-gallyl derivatives: 3-O-galloyl silybin A (SGA) and 3-O-galloyl silybin B (SGB) on cell viability, caspase assessment, total ceramide levels and ceramide-metabolizing enzymes in Hep G2 hepatocarcinoma cell line. Chemical structures of studied compounds are shown in Figure 1.

## Experimental

*Cell lines and reagents*

The human hepatocarcinoma cell line Hep G_2_ was obtained from Institute Pasteur Center for Medical Research. The Hep G_2_ cells were maintained in Dulbecco’s modified Eagle’s medium (DMEM, Promega) supplemented with 10% of FBS (Promega) and 1% penicillin-streptomycin antibiotics (Promega) and were grown at 37 ^°^C in a humidified atmosphere with 5% CO_2_. Silibinin isomers, their 3-O-galloyl derivatives, ceramide and other used reagents were bought from Sigma Chemical Co., St. Louis, MO, USA.

*Cell cultures and preparation of lysates*

The cell line was grown in RPMI-1640 medium supplemented with L-glutamine 2 mM, HEPESNa 25 mM, penicillin 100 U/mL, streptomycin 100 μg/mL and 10% phosphate-buffered saline (FBS) at 37 ^°^C in a humidified atmosphere containing 5% CO_2_. Hep G_2_ cells were seeded at 1 × 106 cells/mL and sub cultured every 2–3 days after 60–80% confluence was reached. To prepare the lysates cells attached to the culture plate were scraped off with a cell scraper and collected in a 1.5 mL tube by centrifugation. The cell pellets were rinsed with PBS, suspended in sterile water, and then lysed by sonication. For each experiment, the cells were treated separately with increasing concentrations of silibinin derivatives (0, 25, 50, 75, 100, 125, 150, 175 and 200 μM) and incubated for 48 h.

*Cell viability*

To measure cell viability, the 3-(4,5-dimethylthiazol-2-yl)-2,5-diphenyl tetrazolium bromide (MTT) colorimetric assay was performed as published (32, 33). Briefly, Hep G_2_ cells were seeded onto 96 flat bottom well plates (50 × 10^3^ cells/well) and grown overnight. After the incubation period with silibinin isomers, cells were washed twice with phosphate-buffered saline solution and incubated with MTT solution at a final concentration of 0.5 mg/mL for 3 h and then lysed in dimethyl sulfoxide. Optical density was measured at 540 nm and the background absorbance measured at 660 nm was subtracted. Each experiment was replicated separately for three times. The results of cell viability are expressed as percentage of control, which was considered to be 100%.

*Caspases activities*

The activities of caspase-3 and 9 were measured using colorimetric substrates. Cells were added to a lysis buffer (100 mM HEPES [pH 7.5], 0.1% CHAPS, 1 mM PMSF, 10 mM MDTT, 1 mM EDTA) and placed on ice for 30 min. After the cells were centrifuged at 10,000 × g for 10 min at 4 ^°^C, 50 μg of protein from the supernatants was added to each of the caspase substrates. The colorimetric substrates for caspase-3 and 9 were Ac-DEVD-pNA (Asp-Glu-Val-Asp-pNA) and Ac-LEHD-pNA (N-acetyl-Leu-Glu-His-Asp-pNA), respectively. After a 2 h incubation, to measure p-nitroanilide absorbance was determined at 405 nm (34).

*Quantifying of total ceramide*

Sample (3 µL of cell extract cell lysate) was mixed with 3 µL of an ACDase assay solution (0.2 M citrate–phosphate buffer, pH 4.5, 0.3 M NaCl, 0.2% Igepal CA-630, 10% FBS, 50 ng/µL ACDase) and incubated at 37 ^°^C for 1 h. The reaction was stopped by adding ethanol (1:5) and centrifuged for 5 min at 13,000 × g. 10 µL of the supernatant was transferred into 20 µL of 25 mM sodium borate buffer (pH 9.0) containing 1.25 mM sodium cyanide and 1.25 mM NDA. The reaction mixture was incubated at 50 ^°^C for 10min, diluted with ethanol (1:4), and centrifuged for 5 min at 13,000 × g. 5 µL of the supernatant was applied to high-performance liquid chromatography (HPLC) for analysis. The HPLC system consisted of Waters 600 S controller, 616 pump, 474 scanning fluorescence detector, 717 auto sampler (Waters, Milford, MA) and BetaBasic-C18 (20 cm × 4.6 mm) column with 3 µM particle size (Thermo Electron, Bellefonte, PA) which was not temperature regulated. All chromatographic procedures were carried out at room temperature using a mobile phase of 90% methanol at a flow rate of 1.0 mL/min. The fluorescent derivatives were monitored at the excitation wavelength of 252 nm and the emission wavelength of 483 nm (35). To calculate the final ceramide contents of the samples, the levels of the endogenous sphingosine (reaction mixture lacking ACDase) were subtracted from the signal obtained in the presence of ACDase. Analysis was based on the principle that one molecule of hydrolyzed ceramide yields one molecule of sphingosine. Standard calibration curves were generated as described above.

*ACDase activity assays*

ACDase activity was measured in intact cells and in cell lysates by fluorogenic assays. For intact cell assays, cells (10,000/well) were seeded into 96-well plates in 10% FBS medium. After 24 h, medium was removed and replaced with 5% FBS medium containing indicated concentrations of silibinin derivatives (controls contained ethanol vehicle). Plates were placed in a tissue culture incubator at 37 ^°^C, 5% CO_2_ for 24 h and cell viability assays were conducted in parallel. Fluorogenic substrate (ethanol vehicle) was then added to a final concentration of 16μM (125 μL final well volume), and the plates were incubated for 3 h at 37 ^°^C, 5% CO_2_. To complete the assays, 50 μL methanol and 100 μL NaIO_4_ (2.5 mg/mL) in 0.1 M glycine buffer with a pH of 10.6 were added and the plates were incubated in a dark place for 2 h at 37 ^°^C. Fluorescence was measured in the UV range (365 nm excitation/410–460 nm emission) using a GloMax® multi-detection system (Promega, Madison, WI). 

To measure ACDase activity in cell lysates, cells were harvested using trypsin/EDTA, washed three times in ice-cold PBS and re-suspended at a concentration of 1 × 106 cells/mL in 0.2 M sucrose. After sonication on ice (microtip, 5–10 s), lysates were centrifuged at 20,000 × g at 4 ^°^C for 15 min to remove debris. Protein in the supernatant was measured using the BCA assay (Pierce Products, Thermo Scientific, Rockford, IL) and bovine serum albumin standard curves. Supernatant protein (60 μg) was added to 96-well plates containing the indicated compounds and sodium acetate–acetic acid buffer, pH 4.5 (25 mM), and incubated for 1 h at 37 ^°^C, in final well volume of 100 μL. Fluorescent substrate was added to a final concentration of 40 μM (125 μL total well volume), and the assay was incubated in the dark at 37 ^°^C for 3 h. Fluorescence was then measured as described above (23).

*NSMase activity assays*

Amplex™ Red NSMase Assay Kit (AAT Bioquest®, Inc. product no: 13620) was used to determine NSMases activity. The kit uses Amplite™ Red as a colorimetric probe to indirectly quantify the phosphocholine produced from the hydrolysis of sphingomyelin by NSMase.

Cells were washed with ice cold PBS and homogenized in neutral lysis buffer (20 mM Tris–HCl pH 7.4, 2 mM EDTA, 5 mM EGTA, 1 mM PMSF, 1% protein cocktail inhibitor and 1 mM sodium orthovanadate) for NSMase assays. Samples were kept on ice 15 min and centrifuged at 14,000 × g for 20 min at 4 ^°^C. 100 µL of each supernatant fraction were incubated at 37 ^°^C for 1hour with working solution. The fluorescence count of produced resorophine in the previous step was measured with a fluorescence micro plate reader by using excitation at 540 nm and emission at 590 nm (36).

*GCS activity assays*

To determine GCS activities, the fluorescent acceptor substrate C6-4-nitrobenzo-2-oxa-1,3-diazole (NBD)-ceramide and a normal-phase HPLC were used. Acceptor substrate, 50 pM of C6-NBD-Cer and 6.5 nM of lecithin were mixed in 100 µL of ethanol and then the solvent was evaporated. Next, 10 µL of water was added and the mixture was sonicated to form liposomes. For the GCS assay, 50 µL of reaction mixture contains 500 µM UDP-Glc, 1mM EDTA, 10 µL of C6-NBD-Cer liposome and 20 µL of an appropriate amount of enzyme in lysis buffer. Addition of conduritol B epoxide (CBE) at 2.5 mM is effective at inhibiting the glycosidase activity. Standard assays were carried out at 37 ^°^C for 1 h. The reaction was stopped by adding 200 µL of chloroform/methanol (2:1, v/v). After a few seconds of vortexing, 5 µL of 500 µM KCl was added and then centrifuged. After the organic phase had dried up, lipids were dissolved in 200 µL of isopropyl alcohol/n-hexane/H_2_O (55:44:1) and then transferred to a glass vial in auto sampler. A 100 µL aliquot of sample was automatically loaded onto a normal-phase column (Intersil SIL 150A-5, 4.6 x 250 mm, GL Sciences, Japan) and eluted with isopropyl alcohol/n-hexane/H_2_O (55:44:1) at a flow rate of 2.0 mL/min. Fluorescence was determined using a fluorescent detector (Hitachi L-7480) set to excitation and emission wavelengths of 470 and 530 nm, respectively. The fluorescent peaks were identified by comparing their retention times with those of standards (37).

*Statistical analysis*

Each experiment was replicated separately for three times. The collected values were analyzed independently, presented as mean ± SD and submitted to statistical evaluation.

The one-way analysis of variance (ANOVA) followed by Tukey’s post-hoc test multiple comparisons was used to indicate the statistical significance of differences between the experimental means. P value< 0.05 was considered significant for all analyses. The data were analyzed using SPSS software (version 19.0). IC50 and EC50 values for each compound were determined by GraphPad Prism software (version 6.07).

## Results

*Cell viability*

**Figure 2:**
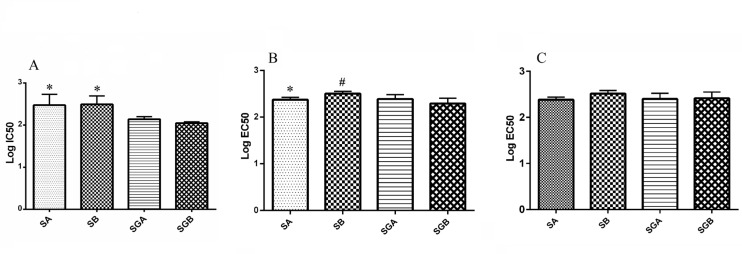
(A) Log IC50 values of silibinin derivatives (silybin A (SA), silybin B (SB), 3-O-galloyl silybin A (SGA) and 3-O-galloyl silybin B (SGB)) for cell viability in Hep G_2_ cell line. All data are expressed as mean ± SD. *Significant difference at P<0.05 in comparison to SGA and SGB.

The effects of our different compounds on cell viability with MTT assay (presented in percentage of control) are shown in Table 1. All studied compounds could decrease this index, among which SA treatment had less inhibitory effect with respect to control. IC50 analysis of studied compounds on this parameter illustrated that SGA and SGB exert more cytotoxic effect in comparison with SA and SB (Figure 2A).

Data also indicated a decreasing pattern in dose dependent manner in which SA, SB, SGA and SGB were more effective respectively. This decrement was significant for all compounds at 125 µM and higher concentrations in respect to control. The highest mortality rate was at 200 µM for all compounds (below 50%) and the lowest level of cell viability was achieved with SGB treatment (approximately around 20% of control). Our data confirmed that gallate moiety intensifies the effectiveness of each silibinin isomer on this parameter. 

*Cell apoptosis*

The activity of caspase isoforms (3 and 9) in response to our studied compounds is exhibited in Tables 2 and 3. The basal level of casapse-3 activity was significantly higher than caspase-9. Both caspases were activated by silibinin derivatives treatment in dose dependent fashion and the activities increased significantly at 100 µM and higher levels for all compounds with respect to control. EC50 analysis indicated that SA is more potent than SB for increasing the activity of caspase-3 and 3-O-galloyl esterification of SB could increase its effect, but this change is not effective for SA. Studied isomers were not different for caspase-9 activity (Figure 2B and 2C).

*Total cellular ceramide levels*

**Figure 3:**
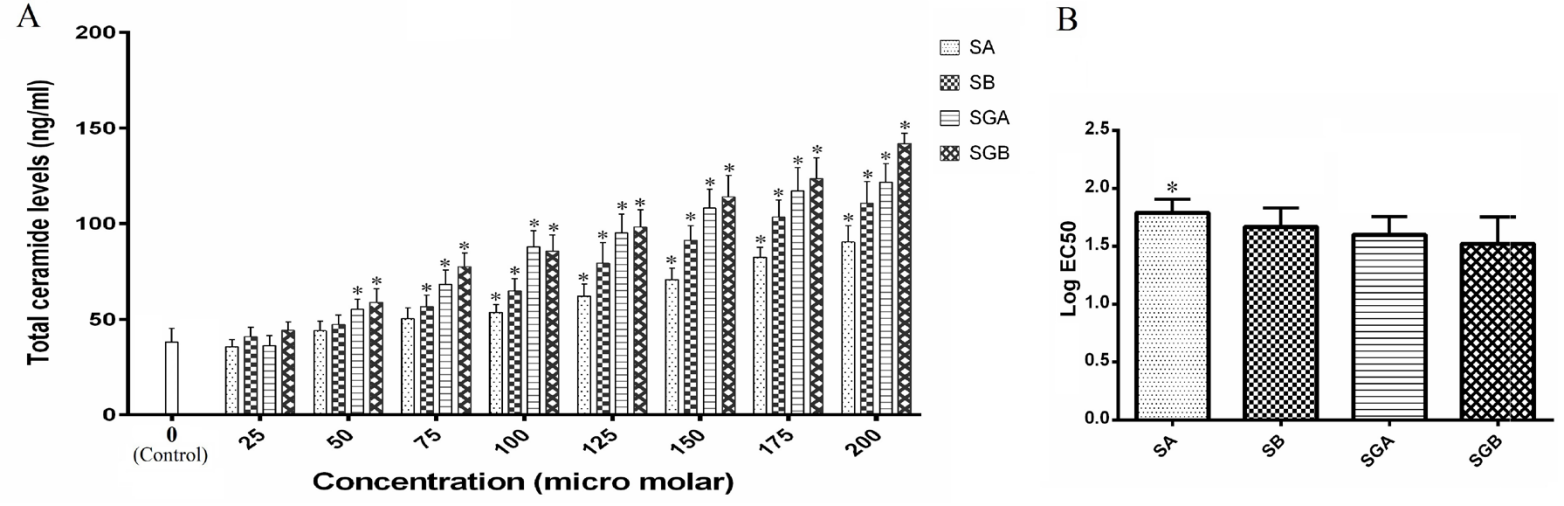
(A) Total cellular ceramide contents (ng/mL) in Hep G_2_ cells after treatment with different concentrations of silibinin derivatives (silybin A (SA), silybin B (SB), 3-O-galloyl silybin A (SGA) and 3-O-galloyl silybin B (SGB)) determined by measuring absorbance of fluorescence substrate (excitation/emission at 252/483 nm). Biological response of each compound evaluated solely in separate cell lysate samples. All data are expressed as mean ± SD; n=3. *Significant difference at P<0.05 compared to control group according to one-way ANOVA, followed by Tukey›s post-hoc test.

The ceramide content levels are presented in Figure 3. Data revealed that cell exposure to silibinin isomers and gallate derivatives leads to an increase in this parameter. Gallate derivatives had stronger ability in ceramide content elevation in comparison with SA and/or SB. There were significant rises in ceramide content in SGB and then SGA treated cells at 50 µM and higher concentrations with respect to control. Maximum ceramide level was obtained by approximately 3.7-fold compared to control in cells under SGB 200 µM (the highest dose) treatment. Moreover, at each treatment concentration, there was no significant difference for ceramide content between SGA and SGB. Also, Data obtained from EC50 analysis revealed a significant potentiating effect for SGB in comparison with SA for elevating total ceramide levels (Figure 3B).

*Activity of ACDase*

**Figure 4:**
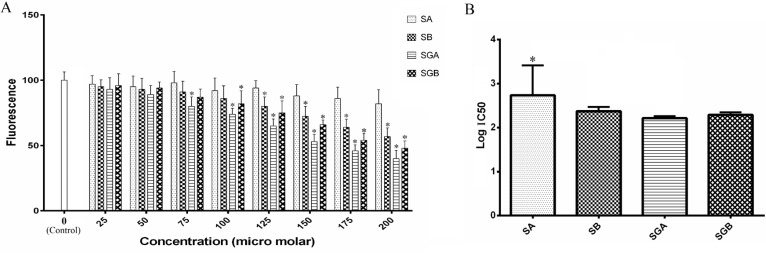
(A) Acid ceramidase activity of Hep G_2_ cells extract treated with different concentrations of silibinin derivatives (silybin A (SA), silybin B (SB), 3-O-galloyl silybin A (SGA) and 3-O-galloyl silybin B (SGB)) determined by measurement the fluorescence (365 nm excitation/410-460 nm emission). All data are presented as mean ± SD; n=3. *Significant difference at P<0.05 compared to control group according to one-way ANOVA, followed by Tukey›s post-hoc test

ACDase activity and IC50 analysis are demonstrated in Figure 4. SGA, SGB and then SB at 125 µM and higher concentrations markedly inhibited the enzyme activity in cell extract compared to control. Cell exposure to SA could not alter ACDase activity throughout the concentration range and we found only 18% inhibition at 200 µM level of treatment. Furthermore, the effect of SGA was statistically higher than SA in the basis of IC50 analysis (Figure 4B).

*Activity of NSMase*

**Figure 5:**
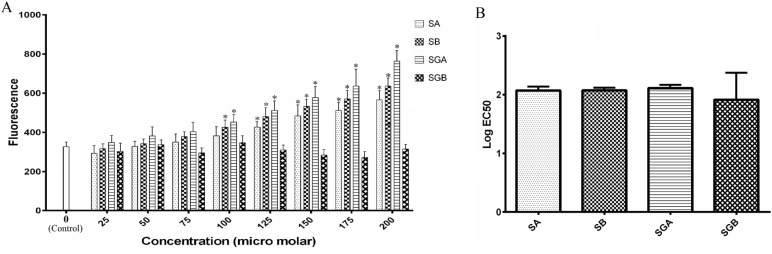
**(A)** The activity of neutral sphingomyelinase (NSMase) in Hep G_2_ cells treated with different concentrations of silibinin derivatives (silybin A (SA), silybin B (SB), 3-O-galloyl silybin A (SGA) and 3-O-galloyl silybin B (SGB)) by fluorescence determination of resorophine (excitation/emission at 540/590 nm). All data are shown as mean ± SD; n=3. *Significant difference at P<0.05 compared to control group according to one-way ANOVA, followed by Tukey›s post-hoc test.

NSMase activity of cell extract in response to treated compounds is shown in Figure 5. Among the studied materials, SGB did not exert any significant change in NSMase activity with respect to control. However, there was a considerable increase in this parameter when the cells were treated with 125 µM and higher concentrations of SA, SB and SGA in comparison with control. The enzyme activity intensified in an increasing pattern by treatment with SA, SB and SGA respectively and reached to the greatest level at 200 µM. However, there was no significant difference between SA and SB at investigated concentrations. On the basis of EC50 analysis, there was no statistical significance between studied compounds (Figure 5B). 

*Activity of GCS*

**Figure 6.**
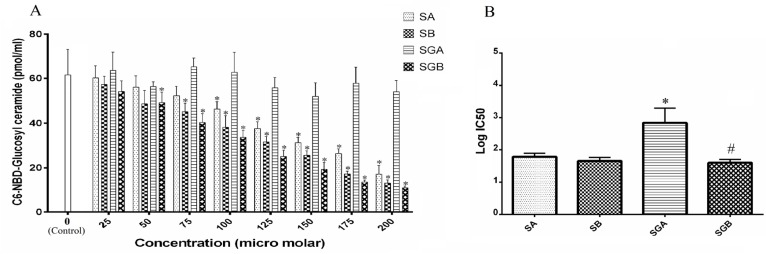
A) The activity of glucosyl ceramide synthase (GCS) in Hep G_2_ cells treated with different levels of silibinin derivatives (silybin A (SA), silybin B (SB), 3-O-galloyl silybin A (SGA) and 3-O-galloyl silybin B (SGB)) by determining concentration of C6-NBD-Glucosyl ceramide considered as GCS activity (excitation/emission at 470/530 nm). All data are shown as mean ± SD; n=3. *Significant difference at P<0.05 compared to control group according to one-way ANOVA, followed by Tukey›s post-hoc test

GCS activity was determined as described in Figure 6. Data did not indicate any significant changes with SGA treatment on GCS activity even at 200 µM. However, SGB, SA and SB caused dose dependent inhibition on this enzyme index. The order of inhibitory effectiveness was: SGB>SB>SA. SA and SB differed significantly at 150 µM and higher treatment concentrations. GCS activity reached to 18% of control after cell exposure to SGB 200 µM that was the lowest among all compounds. The IC50 statistical analysis also confirmed these findings (Figure 6B).

(B) Log EC50 values of studied compounds on caspase-3 activity in Hep G_2_ cell line. All data are expressed as mean ± SD. *Significant difference at P<0.05 in comparison to SB. #Significant difference at P<0.05 in comparison to SGB.

(C) Log EC50 values of studied compounds on caspase-9 activity in Hep G_2_ cell line. All data are expressed as mean ± SD. There was no statistically significant difference between the studied compounds.

(B) Log IC50 values of studied compounds for ACDase activity on Hep G_2_ cell line. All data are expressed as mean ± SD. *Significant difference at P<0.05 in comparison to SGA compound

**Table 1:** The cell viability of Hep G_2_ cells as percent of control after 48 h treatment with different levels of silibinin derivatives (silybin A (SA), silybin B (SB), 3-O-galloyl silybin A (SGA) and 3-O-galloyl silybin B (SGB)) determined by the MTT assay. Biological response of each compound evaluated solely in separate cell lysate samples. All data are shown as mean survival relative to the untreated control ± SD; n = 3. *Significant difference at P<0.05 compared to control group according to one-way ANOVA, followed by Tukey›s post-hoc test

**Concentrations** **(micro molar)**	**Cell viability (% of control)**			
	**SA**	**SB**	**SGA**	**SGB**
0 (Control)	100 + 11.4			
25	97 + 10.0	94 + 10.3	96 + 10.5	91 + 10.0
50	95 + 10.3	89 + 10.5	84 + 9.3^*^	78 + 9.5^*^
75	89 + 11.4	81 + 10.7^*^	73 + 10.7^*^	65 + 8.6^*^
100	83 + 9.5	70 + 10.0^*^	63 + 8.9^*^	56 + 7.7^*^
125	71 + 8.3^*^	60 + 7.9^*^	52 + 8.1^*^	49 + 6.9^*^
150	63 + 7.4^*^	51 + 7.6^*^	44 + 6.0^*^	35 + 5.1^*^
175	52 + 6.0^*^	45 + 5.1^*^	38 + 5.7^*^	29 + 4.3^*^
200	47 + 5.5^*^	40 + 6.0^*^	33 + 5.1^*^	24 + 3.4^*^

**Table 2:** The caspase-3 activity of Hep G_2_ cells after 48 h treatment with various concentrations of studied compounds (silybin A (SA), silybin B (SB), 3-O-galloyl silybin A (SGA) and 3-O-galloyl silybin B (SGB)) determined by measuring optical absorbance of p-nitroanilide. All data are shown as mean ± SD; n = 3. *Significant difference at P<0.05 compared to control group according to one-way ANOVA, followed by Tukey›s post-hoc test

**Concentrations** **(micro molar)**	**Caspase-3 (ΔOD 405nm)**			
	**SA**	**SB**	**SGA**	**SGB**
0 (Control)	0.38 + 0.043			
25	0.45 + 0.055	0.34 + 0.050	0.35 + 0.038	0.45 + 0.065
50	0.58 + 0.080	0.45 + 0.060	0.46 + 0.062	0.56 + 0.070
75	0.65 + 0.098^*^	0.58 + 0.076	0.58 + 0.070	0.62 + 0.081^*^
100	0.79 + 0.107^*^	0.53 + 0.065	0.67 + 0.084^*^	0.78 + 0.095^*^
125	0.91 + 0.133^*^	0.62 + 0.095^*^	0.83 + 0.114^*^	0.98 + 0.128^*^
150	0.98 + 0.148^*^	0.73 + 0.105^*^	0.97 + 0.134^*^	1.21 + 0.160^*^
175	1.12 + 0.164^*^	0.82 + 0.130^*^	1.12 + 0.147^*^	1.53 + 0.209^*^
200	1.06 + 0.152^*^	0.79 + 0.096^*^	1.26 + 0.164^*^	1.63 + 0.221^*^

**Table 3:** The caspase-9 activity of Hep G_2_ cells after 48 h treatment with various concentrations of studied compounds (silybin A (SA), silybin B (SB), 3-O-galloyl silybin A (SGA) and 3-O-galloyl silybin B (SGB)) determined by measuring optical absorbance of p-nitroanilide. All data are shown as mean ± SD; n = 3. *Significant difference at P<0.05 compared to control group according to one-way ANOVA, followed by Tukey›s post-hoc test

**Concentrations** **(micro molar)**	**Caspase-9 (ΔOD 405nm)**			
	**SA**	**SB**	**SGA**	**SGB**
0 (Control)	0.26 + 0.034			
25	0.41 + 0.060^*^	0.30 + 0.048	0.23 + 0.029	0.30 + 0.048
50	0.50 + 0.089^*^	0.41 + 0.055^*^	0.40 + 0.053^*^	0.41 + 0.053^*^
75	0.61 + 0.102^*^	0.47 + 0.070^*^	0.55 + 0.070^*^	0.46 + 0.046^*^
100	0.73 + 0.110^*^	0.56 + 0.067^*^	0.86 + 0.136^*^	0.53+ 0.077^*^
125	0.84 + 0.133^*^	0.60 + 0.083^*^	0.69 + 0.103^*^	0.70 +0.093^*^
150	0.96 + 0.143^*^	0.67 + 0.105^*^	0.80 + 0.124^*^	0.89 + 0.108^*^
175	1.10 + 0.164^*^	0.81 + 0.126^*^	1.19 + 0.147^*^	1.18 + 0.192^*^
200	1.16 + 0.147^*^	0.89 + 0.114^*^	1.30 + 0.167^*^	1.34 + 0.207^*^

## Discussion

Silibinin as a polyphenolic flavonoid has been introduced as a chemothrerapeutic agent in many studies (38). As a new aspect, our research evaluates the role of two diastereoisomers of silibinin and their gallate derivatives on cell cytotoxicity, apoptosis and ceramide metabolism pathway.

In this study, the *in-vitro* application of silibinin derivatives had cytotoxic effects on Hep G_2_ human liver carcinoma cells with different intensities as tested by MTT assay. Data confirmed that cell viability decreased in a dose dependent fashion with silibinin derivatives in which SA, SB, SGA and SGB were more potent respectively. Galloyl esterification ordinarily could enhance the cytotoxic effect of silibinin stereoisomers. In accordance to our findings, MTT evaluation of silibinin on SKBR3 breast cancer cell line recorded by Mahmoodia *et al*. and Provinciali *et al.* on mammary tumors of transgenic mice. Their data showed significant concentration and time dependent inhibitory effects on cell growth (39, 40). Furthermore, Davis-Searles *et al.* denoted that among commercial silymarin extracts, four compounds namely SA, SB, isosilybin A and isosilybin B had the most consistent anti-proliferative effects in different human carcinoma cell lines (41). 

Cytotoxic response could be mediated by apoptosis inducing and activation of proteolytic enzymes known as caspases. Caspases are critical components of the execution phase of cell death in most forms of apoptosis (42). On this basis, we checked two main caspases activities in response to our treatments. Both studied caspases potentiated with silibinin derivatives in a dose-dependent manner. based these results, we suggest that the reinforcing effect of silibinin in Hep G_2_ hepatocarcinoma cell line could be significantly enhanced through galloyl esterification in some situations. In accordance to our data, study on K562 leukemia cells by Zhong *et al.* and a report on ovarian cancer cells by Fan *et al.,* demonstrated that silymarin caused activating effect on both mentioned caspases and subsequently apoptosis (10, 43).

In recent years, the sphingolipid ceramide has been described as a key pathway in apoptosis inducing in many cell types (17, 44). This second messenger is also involved in cell cycle regulation, proliferation and differentiation (45). Ceramide is a ubiquitous bioactive lipid and is involved on conversion of cytostatic to cytotoxic end-point in cancerous cells (46).

Accordingly, we evaluated the effects of silibinin stereoisomers and their galloyl derivatives on total ceramide contents within treated cells. Regarding the result of our findings, all studied compounds elevated the total ceramide concentrations and this effect was enhanced by galloyl esterification. In related to our study, it has been confirmed that in several cancerous cells such as prostate cancer, pro-apoptotic events by flavonoids were most likely mediated by *de novo* synthesis of ceramide or inhibition of ceramide degradation (47). As an experimental document, it has been observed that administration of exogenous ceramide analogs or increased level of total intracellular ceramide is associated with caspase-independent apoptosis in several types of cells (45). In addition, other results suggest that ceramide may act as a mediator of apoptosis by activating or inducing of caspases (48). Therefore, we concluded that the involvement of ceramide in the observed cytotoxic effects induced by silibinin derivatives.

The enzymes associated with ceramide metabolism should be effective and ideal target in the study of cancer chemo-preventive agents (23, 49). Because of broad spectrum effects of silibinin on diverse cell signaling pathways and considering the important role of ceramide on these aspects, the investigation of silibinin effects on ceramide metabolism may be useful (50). In the current study, we evaluated the effects of silibinin stereoisomers and their gallate derivatives on ceramide metabolism related enzymes.

ACDase is involved in ceramide degradation to sphingosine backbone and acetyl group. It occupies an important place in control of ceramide levels in cancer cell responses to different exogenous factors (24). With regard to this part of ceramide metabolism pathway, our evaluation demonstrated that SB had moderate inhibitory effect on ACDase although SA was inert. On the other hand, galloyl substitution of these stereoisomers significantly potentiated this inhibitory effect. In agreement to our result, another report on response of cancerous cells to chemotherapy confirmed that inhibition of ACDase could reduce cell proliferation and migration (26).

NSMase is another enzyme which counterparts to ceramide metabolism by degradation of sphingomyeline to ceramide. This enzyme is activated in response to many extracellular stimuli such as some chemotherapeutic agents (51). There is related evidence to our findings that indicates flavonoids could alter ceramide levels via acting on NSMase activity in hepatocyte under toxic conditions (52). We showed that the activity of NSMase increases with SA, SB and SGA.

GCS is an enzyme which catalyzes the first step of glycolipid biosynthesis by transferring of glucose to ceramide (18). The result of our study denoted that both SA and SB could reduce the activity of GCS. However, the galloyl esterification of SB made it a more potent inhibitor but this alternation for SA led to creating an inert substance. It has been recognized that suppression of GCS in cancer cells, led to the amplification of cytotoxic response to chemotherapy (53).

Totally, we observed that SGB had the most accumulating effect on ceramide in comparison with other compounds. This effect was occurred through intensive inhibitory pattern on GCS activity and simultaneously moderate retarding effect on ACDase. In this study SGA as second ceramide accumulator exerted great inhibitory effect on ACDase and then on GCS activities. Accordingly, we hypothesized that GCS played more important role among the studied ceramide-metabolizing enzymes in Hep G_2_ cells. 

In our study, SB and SA were at third and fourth grades on cytotoxicity induction and ceramide generation. This effect could be attributed to a more stimulatory effect on NSMase and a moderate inhibition of GCS and ACDase by SB with respect to SA. 

Our study was the first in its kind that investigated silibinin, its galloyl substituted and its isomers on ceramide metabolism in Hep G_2 _cell line. There are several studies on the different biological aspects of these compounds. In human bladder and colon cancer cell lines, 7-O-galloyl silibinin demonstrated better growth inhibitory effects compared to silibinin, and other silibinin derivatives (54). Another report revealed that the presence of mono galloyl moiety of natural silybin (a mixture of both isomers: SA and AB) leads to increase in their anti-angiogenic activities (55). In addition, two isomers of silibinin: SA and/or SB exhibit partially poor anti-angiogenic activities with regard to galloyl-esterified form of silibinin itself (56). 

In conclusion, silibinin isomers and galloyl substituted forms had cytotoxic effects in Hep G_2_ cells that were raised from ceramide up-regulation. Galloyl substitution intensified effects of silibinin on total ceramide levels and ceramide*-*metabolizing enzymes, particularly GCS and ACDase. The increased anti-cancer effects shown by these galloyl derivatives may suggest new chemical strategies to potentiate the sibilinin anti-cancer features.

## Abbreviations:

ACDase, Acid ceramidase; DMEM, Dulbecco’s modified Eagle’s medium; GCS, glucosyl ceramide synthase; MTT, 3-(4,5-dimethylthiazol-2-yl)-2,5-diphenyl tetrazolium bromide; NSMase, Neutral sphingomyelinase; RPMI, Roswell Park Memorial institute; SA, silybin A; SB, silybin B; SGA, 3-O-galloyl silybin A; SGB, 3-O-galloyl silybin B; SPT, serine palmitoyl transferase.
